# Frequency-Specific Blood Oxygen Level Dependent Oscillations Associated With Pain Relief From Ankle Acupuncture in Patients With Chronic Low Back Pain

**DOI:** 10.3389/fnins.2021.786490

**Published:** 2021-12-07

**Authors:** Anfeng Xiang, Meiyu Chen, Chuan Qin, Jun Rong, Can Wang, Xueyong Shen, Sheng Liu

**Affiliations:** ^1^School of Acupuncture-Moxibustion and Tuina, Shanghai University of Traditional Chinese Medicine, Shanghai, China; ^2^The First Rehabilitation Hospital of Shanghai, School of Medicine, Tongji University, Shanghai, China; ^3^Department of Sports Rehabilitation, Zhejiang Integrated Traditional and Western Medicine Hospital, Zhejiang, China

**Keywords:** ankle acupuncture stimulation, immediate analgesia, resting-state brain activity, insular, cerebellum

## Abstract

**Objective:** Recent advances in brain imaging have deepened our knowledge of the neural activity in distinct brain areas associated with acupuncture analgesia. However, there has not been conclusive research into the frequency-specific resting-state functional changes associated with acupuncture analgesia in patients with chronic pain. Here, we aimed to characterize changes across multiple frequencies of resting-state cortical activity associated with ankle acupuncture stimulation (AAS) in patients with chronic low back pain (CLBP) and healthy controls.

**Methods:** Twenty seven patients with CLBP and Twenty five age- and gender-matched healthy volunteers were enrolled in the study. Participants received tactile sham acupuncture (TSA) and AAS, respectively. The whole-brain amplitude of low-frequency fluctuation (ALFF) in the range 0.01–0.25 Hz was assessed for changes associated with each intervention. Further, a visual analog scale (VAS) was used to collect subjective measures of pain intensity in patients. Linear mixed-effect modeling (LME) was used to examine the mean ALFF values of AAS and TSA between patients and healthy controls.

**Results:** The ALFF was modulated in the default mode network (an increase in the medial prefrontal cortex, and a decrease in the cerebellum/posterior ingulate/parahippocampus, *P* < 0.01, corrected) in both patients and controls. Decreased ALFF in the bilateral insular was frequency-dependent. Modulations in the cerebellum and right insular were significantly correlated with VAS pain score after AAS (*P* < 0.01).

**Conclusion:** Hence, frequency-specific resting-state activity in the cerebellum and insular was correlated to AAS analgesia. Our frequency-specific analysis of ALFF may provide novel insights related to pain relief from acupuncture.

## Introduction

Chronic low back pain (CLBP) affects 60–80% of people at some point in their lifetime and has become one of the most common reasons for visiting a physician (Hoy et al., 2010; Urits et al., 2019). Nearly a third of people who seek treatment for low back pain (LBP) will experience persistent back pain for 1 year after the acute period (Harrisson et al., 2017). Prolonged loss or impairment of function caused by LBP often has an economic impact on the patient, including treatment costs, disability payments, and reduction or loss of productivity. LBP is associated with structural, neurochemical, and functional changes in the brain, in regions such as the thalamus, the anterior insula, and the somatosensory cortex (Gao et al., 2016; Konno and Sekiguchi, 2018). Complex processes induced by peripheral and central sensitization following the experience of pain may be involved in the transition from acute to chronic LBP.

A growing body of evidence supports the use of acupuncture as an effective treatment for acute and chronic pain, including headaches, neck pain, osteoarthritis pain, and LBP (Furlan et al., 2005; Berman et al., 2010; Wang et al., 2021). The efficacy of acupuncture as an analgesic has also been verified using incision pain, and inflammatory and neuropathic pain-related behavioral tests in animal studies (Zhang et al., 2014, 2021). Nevertheless, the neural mechanisms underlying the analgesic effects of acupuncture have not yet been fully investigated. Functional magnetic resonance imaging (fMRI) provides a useful technique to investigate the mechanisms by which processes in the central nervous system are modulated by acupuncture. fMRI studies on acupuncture at commonly used acupoints have demonstrated significant functional response in many brain regions associated with acupuncture analgesia, such as the prefrontal cortex, the limbic system, the paralimbic and subcortical gray structures, and the cerebellum (Hui et al., 2000; Napadow et al., 2005; Wang et al., 2013).

Frequency-specific blood oxygen level dependent (BOLD) signal oscillations measured using resting-state fMRI (rs-fMRI) are of increasing interest, and the nature of this frequency-specificity has many biological interpretations. Studies reporting specific frequencies of BOLD signal oscillations indicated that each different frequency band contributes uniquely to brain network integration in terms of physiological and pathological activities (Hipp and Siegel, 2015; He et al., 2016). Oscillations within the 0.01–0.25 Hz range can be consistently divided into the intrinsic frequency bands, norm-1 (0.01–0.1 Hz), norm-2 (0.01–0.08 Hz), slow-5 (0.01–0.027 Hz), slow-4 (0.027–0.073 Hz), slow-3 (0.073–0.198 Hz), and slow-2 (0.198–0.25 Hz) (Lou et al., 2020). These frequency bands have been shown to be highly reproducible and independent of the fMRI sampling rate. It has been proposed that different frequency bands signify different physiological changes (Yan et al., 2020). For example, BOLD signal oscillations lower than 0.02 Hz were typically observed in the putamen and higher-frequency oscillations (>0.08 Hz) in limbic areas (Zuo et al., 2010). As such, a frequency-specific approach may provide more information than conventional approaches (relying predominantly on oscillations < 0.1 Hz) to help interpret localized BOLD changes associated with acupuncture stimulation in patients with LBP. To our knowledge, no previous study has assessed the frequency-specific resting-state functional changes associated with acupuncture analgesia in LBP patients.

In the present study, we explored the contribution of different frequency bands to changes in resting-state BOLD fMRI associated with the administration of acupuncture in LPB patients. To detect the frequency-specific functional changes that may play an important role in acupuncture analgesia, we assessed BOLD oscillations in frequency bands (0.01–0.1 Hz, 0.01–0.08 Hz, 0.01–0.027 Hz, 0.027–0.073 Hz, 0.073–0.198 Hz, and 0.198–0.25 Hz) to compare resting-state activity in LBP patients with that of healthy controls. A painless acupuncture technique, ankle acupuncture stimulation (AAS), was used as the intervention method. AAS is a type of subcutaneous acupuncture that was developed in the 1970s (Zhang, 1997). It has been shown that AAS not only has a measurable analgesic effect in numerous conditions associated with pathological pain such as low back pain, cancer, and postoperative recovery (Zeng et al., 2014; Zhu et al., 2014), but also significantly improves the pain threshold of healthy individuals (Marra et al., 2011). AAS requires the insertion of a single needle near the ankle without inducing any localized sensation of the needle. Its stimulation is mild and comprises merely a tactile sensation on the skin. Therefore, AAS may be helpful in investigating patterns of brain activity specifically associated with acupuncture analgesia by excluding or minimizing the possibility of confounding factors such as somatosensation induced by Deqi needle manipulation in traditional acupuncture methods.

## Materials and Methods

### Participants

The study protocol was formulated in accordance with the Declaration of Helsinki and approved by the ethical committee of the Center of Cognitive and Brain Disorders of Hangzhou Normal University (20190102). Our present study was registered on www.chictr.org.cn (Identifier: ChiCTR1800020029). Participants gave written informed consent before undergoing screening.

Patients were assessed by an experienced rehabilitation physician according to the following criteria. The inclusion criteria of patients were as follows: (1) right-handed adults of any gender aged 18 to 65 years; (2) a history of low back pain for 6 months or more; (3) an initial self-reported pain intensity evaluated using the visual analog scale (VAS, 0–10) of at least 4 points; (4) able to understand and complete the consent form and clinical assessment questionnaires without assistance. The inclusion criteria of healthy subjects were as follows: (1) right-handed, age- and gender-matched adults; (2) no history of pain or relevant serious disease; (3) able to understand and complete the consent form without assistance.

The exclusion criteria of patients and healthy subjects were as follows: (1) the presence of another cause of pain unrelated to LBP; (2) having taken any medication or undergone physical therapy in the past week; (3) any other serious illnesses or neuropsychiatric diseases; (4) any contraindications for undergoing MRI, including the presence of metal implants, cardiac pacemakers, or claustrophobia; (5) any contraindications for undergoing acupuncture, such as pregnancy or a tendency to bleed easily; (6) a history of sleep deprivation or women who were experiencing their menstrual period; (7) having consumed coffee or alcohol in the 10 h prior to the MRI scan. Participants were free to withdraw at any time. To ensure data quality, any individual who moved their head by more than 1 mm or 2° during the scan was excluded from the analyses.

### Randomization and Blinding

The order of AAS and TSA runs was pseudo-randomized across subjects to reduce order effects. In order to maximize washout of acute stimulation effects, these two runs (AAS or TSA) were separated by structural scans which lasted 15 min. In the present study, participants were acupuncture naïve and were informed that there would be “different forms” of acupuncture during fMRI. In addition, the needles used for AAS and TSA were identical. Our preliminary test showed that the sensations of AAS and TSA were similar. The locations of needle stimulation were taped with medical adhesive tape. Participants lay supine in the scanner with their vision of distal body regions blocked by the MRI head coil, preventing them from viewing the intervention occurring at their periphery. Participants could be blinded to the order of AAS and TSA. Blinding success was validated at the end of the study.

### Ankle Acupuncture Stimulation Stimulation

**FIGURE 1:**
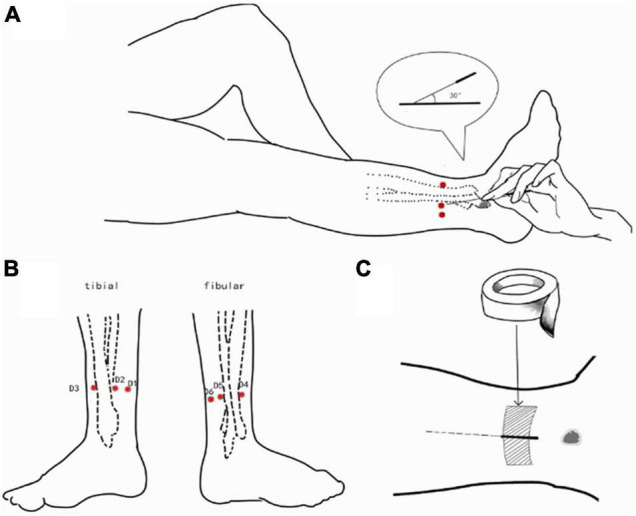
AAS was performed at the left lower 5th AAS zone. **(A)** The acupuncturist inserted the needle into the skin at an angle of about 30°. The needle was angled horizontally into the subcutaneous layer and inserted toward the direction of the knee about 35 mm; **(B)** the needle insertion point (three-finger widths above the lateral malleolus, near the posterior edge of the fibula and adjacent tendons); **(C)** the locations of needle stimulation were taped with medical adhesive tape.

AAS and TSA were performed by the same experienced acupuncturist using disposable silver acupuncture needles (0.35 × 40 mm; Zhongyan Taihe Brand, Beijing, China). The left lower 5th AAS zone was selected as the needle insertion point, with the exact location about three-finger widths above the lateral malleolus, near the posterior edge of the fibula and adjacent tendons (Figure 1). The acupuncture method used was based on that described in “Wrist-Ankle Acupuncture” (Zhang, 1997). Briefly, the participant laid in a supine position and exposed the skin of the lateral side of the left leg. After sterilizing the skin and needle, the acupuncturist inserted the needle into the skin at an angle of about 30°. The needle was angled horizontally into the subcutaneous layer and inserted toward the direction of the knee about 35 mm. Participants did not feel any sensation during AAS if this procedure was correctly conducted. The locations of needle stimulation were taped with medical adhesive tape.

During TSA, participants exposed the skin on the lateral side of the left leg, and the stimulus was administered to the same location as in AAS. Instead of inserting a needle into the skin, the needle was gently placed against the skin and held there for 5 s without penetrating the skin. All other procedures were identical to those used in AAS.

### Functional Magnetic Resonance Imaging Scanning Procedure

The study was conducted in the MRI room of the Center of Cognitive and Brain Disorders, Hangzhou Normal University. Participants were scanned on a GE-750, 3 T MRI system with an 8-channel head coil. The participants wore earplugs and lay in a supine position on the scanner bed. Sponge padding was used to minimize head movement. During the scanning process, participants were told to keep their eyes closed, stay awake, and try not to think of anything.

The fMRI acquisition used a gradient echo (GRE) echo-planar imaging (EPI) sequence with the following parameters: number of slices = 43, acquired interleaved, matrix size = 64 × 64, repetition time (TR) = 2,000 ms, echo time (TE) = 30 ms, flip angle (FA) = 90°, slice thickness = 3.2 mm, voxel size = 3.4 × 3.4 × 3.2 mm^1^. A total of 240 whole-brain volumes were obtained. A 3D T1-weighted structural image was acquired using a spoiled gradient echo (SPGR) sequence, as follows: number of slices = 176, matrix size = 256 × 256, TR = 8,100 ms, TE = 3.1 ms, FA = 8°, slice thickness = 1 mm, voxel size = 1 × 1 × 1 mm^3^, inversion time = 450 ms, and bandwidth = 31.25 Hz. Each participant received one functional image scan following administration of either the TSA or AAS (all participants received both TSA and AAS). The structural image was acquired between the two functional runs.

**FIGURE 2:**
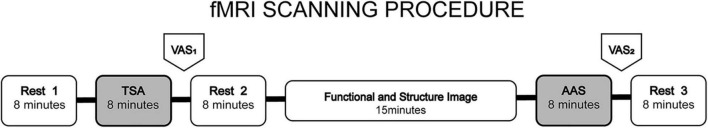
fMRI scanning paradigm. The order of TSA and AAS runs was pseudo-randomized across subjects. After TAS or AAS stimulation run (needles withdrawal), rest runs were conducted. During rest runs, subjects were asked to lie quietly, keep their eyes closed and stay awake. The TSA and AAS portions of the scan session were separated by functional and structure image scan lasting 15 min.

fMRI scanning paradigm is shown in Figure 2. Similar to the study of Dhond et al. (2008), the order of TSA and AAS runs was pseudo-randomized across subjects. The TSA and AAS portions of the scan session were separated by functional and structure image scan lasting 15 min. 8 min rest run was completed before, and after each stimulation run. During rest runs, subjects were asked to lie quietly, keep their eyes closed and stay awake.

### Pain Assessment

A self-reported VAS was used to evaluate the current pain intensity experienced by each participant immediately after the TSA and AAS of the scan session. VAS rating was accomplished by the use of a custom-built MR-compatible handheld rotating knob, which was connected to a visual display projecting an intensity scale. The knob was set to traverse the entire rating range with minimal thumb finger twisting. The device was held in the right arm of participant. The VAS took < 1 min to complete. The scale was comprised of a 10 cm horizontal line, extending from “0” on the left indicating no pain, to “10” on the right indicating the most severe pain imaginable. Participants were instructed to mark a “×” on the line according to the pain intensity they experienced currently. The length (in centimeters) from”0” to the mark was recorded as their pain score.

### Functional Magnetic Resonance Imaging Data Analysis

MRI data were processed in MATLAB (R2014), using SPM12^2^ and RESTplus^3^. The raw data were preprocessed as follows: the first 10 brain volumes were removed, slice timing and head motion correction were applied, functional and structural images were realigned, the structural image was segmented, and all data were spatially normalized into Montreal Neurological Institute (MNI) space, resampled at 3 × 3 × 3 mm, and spatially smoothed using a Gaussian kernel of 6 mm full-width half-maximum (FWHM). Finally, the statistical model included regression of covariates (including Friston-24 head movement parameters, and white matter and cerebrospinal fluid time courses) and removal of the linear trend.

All BOLD signal fluctuations below the Nyquist frequency (0.25 Hz in the present study) were delineated into six frequency bands: norm-1 (0.01–0.1 Hz), norm-2 (0.01–0.08 Hz), slow-5 (0.01–0.027 Hz), slow-4 (0.027–0.073 Hz), slow-3 (0.073–0.198 Hz), and slow-2 (0.198–0.25 Hz) (Buzsáki and Draguhn, 2004). The whole-brain level of Amplitude of Low-Frequency Fluctuation (ALFF) was calculated within each frequency band. These ALFF values were converted to z-score standardized values (zALFF) before statistical analysis.

### Statistical Analyses

Demographic data and VAS pain intensity scores were visualized using Graphpad prism 8^4^. Descriptive statistics were presented as mean ± standard deviation. Here we used a linear mixed-effect modeling (LME) to examine the mean ALFF values of AAS and TSA between patients and healthy controls. LME is a flexible modeling approach that handles complex experimental designs and has been widely used in fMRI statistical analysis (Chen et al., 2013; Jo et al., 2021; Yang et al., 2021). The difference in VAS score between TSA and AAS conditions was determined using a two-tailed paired *t*-test and significance considered at a threshold of 0.05. The main effects of group (patients vs. healthy controls) and condition (AAS vs. TSA), and any interaction between the two were determined using a mixed-effects analysis in DPABI (V4.1)^3^ toolbox. Results were corrected based on Gaussian random field theory (GRF) (*z* > 2.3, *p* < 0.01, cluster > 20 voxels). *Post hoc* analyses were performed when a significant interaction effect was detected. Associations between zALFF at the cluster peak coordinates and VAS scores were explored by calculating the Pearson correlation coefficient.

## Results

### Participants

Fifty two participants (27 patients with CLBP and 25 volunteers) were recruited from Zhejiang Integrated Traditional and Western Medicine Hospital. 12 patients dropped out during the experimental period. Three participants could not tolerate the scanning session. Two participants were not imaged during acupuncture stimuli due to claustrophobia. Three participants terminated the study due to excessive fear. One subject was removed for suspected sub-clinical neuropathy. Three participants voluntarily withdrew. A final total of 40 patients (20 in the CLBP and 20 in the healthy subjects group) completed all the clinical assessments and imaging scans. In addition, one patient and one control were excluded because of severe head movement during fMRI. Therefore, data from 19 (*n* = 12 men) patients and 19 (*n* = 11 men) healthy subjects comprised the final dataset for analysis. All the participants reported no obvious needle sensation during acupuncture. The basic characteristics of patients with CLBP were as follows (Table 1): age 46 ± 7 years, height 165.4 ± 7.9 cm, weight 64.05 ± 5.63 kg. There were no significant differences in the above demographic measures between the two participant groups (*P* > 0.05), but duration of lower back pain in patients was 9.0 ± 7.7 years.

**TABLE 1:** Characteristics of included participants.

	Group		*t*/χ^2^	*P-*value
	Healthy control	Patient		
Gender (M/F)	11/8	12/7	0.11	0.74
Age (years)	38.95 ± 15.45	46.61 ± 7.35	1.66	0.11
Weight (kg)	66.18 ± 10.18	64.05 ± 5.63	0.80	0.43
Height (cm)	168.10 ± 7.33	165.40 ± 7.86	1.11	0.27
Pain duration	–	9.00 ± 7.72	–	–

### Visual Analog Scale Pain Intensity Scores

At a group level, the VAS scores of pain in patients were significantly (*t* = 6.10, *P* = 0.001) lower after AAS (3.50 ± 2.23) in comparison to after TSA (5.64 ± 1.98). Duration of experiencing lower back pain was negatively correlated with the degree to which the VAS pain intensity score differed between the two conditions (*r* = –0.52, *P* = 0.02). All of healthy subjects were scored with VAS ≤ 1 at the two observation points. There was no significant difference noticed in the VAS scores of pain after AAS in comparison to after TSA (*P* > 0.05).

### Multi-Frequency Band Z-Score Standardized Values

**FIGURE 3:**
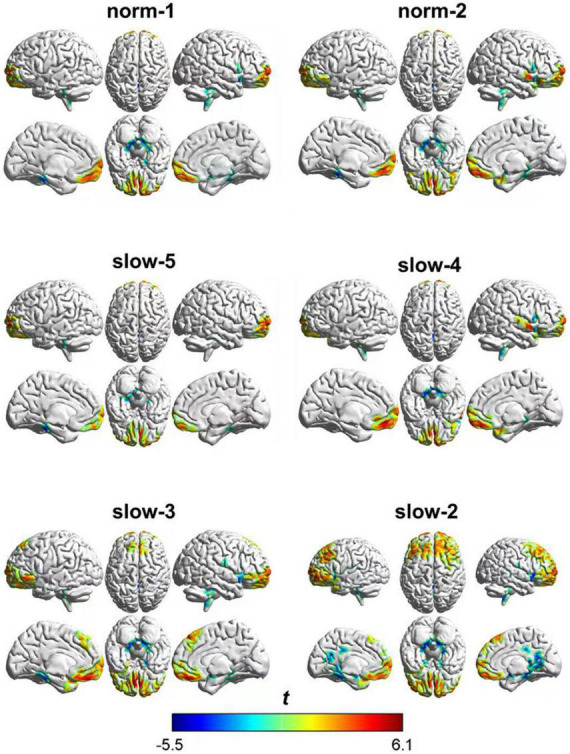
Whole-brain parameter maps showing the main ALFF effects (AAS-TSA).

As shown in Figure 3, at a group level, zALFF following AAS showed significant differences in several brain areas compared with that after TSA. These differences showed both similarities and differences when compared between frequency bands.

As shown in Table 2, zALFF within all frequency bands increased in the medial prefrontal cortex and decreased in the cerebellum, posterior cingulate cortex, and parahippocampal gyrus. For all frequency bands except the slow-5 band, zALFFs decreased in the right insular cortex. Except for the norm-2 and slow-5 bands, zALFF increased in the right superior temporal gyrus. zALFF within norm-2, slow-3, and slow-2 bands decreased in the right precentral and postcentral gyrus, and amygdala. zALFF in the slow-2 band decreased in the left insula, amygdala, and precentral gyrus. Further, zALFF within each frequency band did not show any statistically significant differences with the main effects of participant group or any interaction between group and treatment.

**TABLE 2:** The ALFF results of main effects (AAS-TSA).

Band	Area	BA	Cluster size	*t-*value	MNI coordinate		
					x	y	Z
Norm-1	mPFC	10_‵_ 11	1,222	4.99	–6	54	–21
	Cerebellum, PCC, parahippocampus	27_‵_ 30	655	–5.48	3	–39	–15
	Right insula	13	421	–5.31	39	–6	21
Norm-2	mPFC	10_‵_ 11	1,199	5.21	–6	54	–21
	Right superior temporal gyrus	38	231	4.88	60	9	–6
	Cerebellum, PCC, parahippocampus	27_‵_ 30	572	–5.71	3	–39	–15
	Right insula, precentral gyrus, postcentral gyrus, amygdala	13_‵_ 6_‵_ 24	346	–5.35	39	–6	21
Slow-5	mPFC	10_‵_ 11	1,009	5.06	–30	66	0
	Cerebellum, PCC, parahippocampus	27_‵_ 30	288	–5.06	21	–33	–27
Slow-4	mPFC	10_‵_ 11_‵_ 34	890	5.12	–6	54	–21
	Right superior temporal gyrus	38_‵_ 22	258	5.19	57	9	–9
	Cerebellum, PCC, parahippocampus	30_‵_ 25	454	–5.44	3	–39	–15
	Right insula	13_‵_ 6	253	–4.84	39	–6	21
Slow-3	mPFC, Right superior temporal gyrus	10_‵_ 11_‵_ 38	1,622	6.10	0	39	–24
		8_‵_ 6	301	4.18	9	24	60
	Cerebellum, PCC, parahippocampus	30_‵_ 35	629	–4.75	27	–39	–30
	Right insula, precentral gyrus, postcentral gyrus, amygdala	13_‵_ 4,722_‵_ 3,834_‵_ 6	448	–4.58	51	9	6
Slow-2	mPFC, Right superior temporal gyrus	6_‵_ 8–1,138_‵_ 46	3,327	5.16	12	36	–24
	Cerebellum, PCC, parahippocampus	23_‵_ 2,730	1,358	–5.43	15	–36	–15
	Right insula, precentral gyrus, postcentral gyrus, amygdala	13_‵_ 4,738_‵_ 2,234_‵_ 6	764	–5.12	45	0	3
	Left insula, precentral gyrus, amygdala	13_‵_ 2,234_‵_ 4,144	439	–4.68	–45	–18	3

*BA, Brodmann; MNI, Montreal Neurological Institute; mPFC, medial prefrontal cortex; PCC, posterior cingulate cortex.*

**FIGURE 4:**
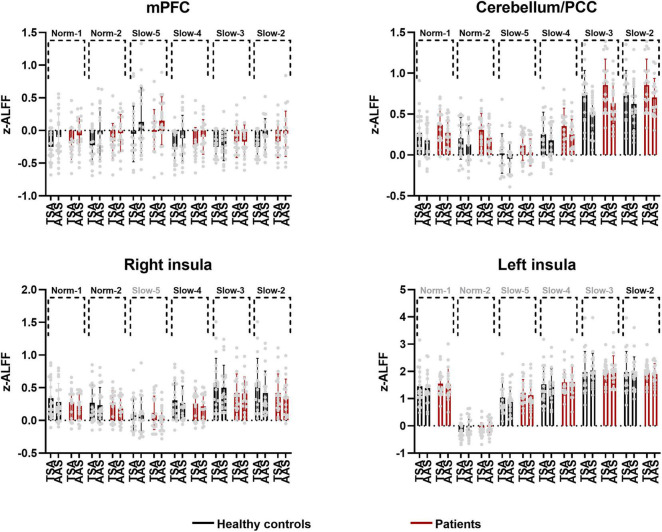
Individual and group values of zALFF across frequency bands in mPFC, cerebellum/PCC, right and left insula (TSA, tactile sham acupuncture; AAS, ankle acupuncture stimulation, frequency bands in gray denote no difference in the present comparison).

The area over which each zALFF frequency band was present was determined. Following this, the area where all frequency bands overlapped was also determined. The peak coordinates of this area were found in the medial prefrontal cortex (mPFC; *x* = –6, *y* = 54, *z* = –21) and cerebellum/posterior cingulate cortex (PCC) (*x* = 0, *y* = –42, *z* = –15). The peak coordinate of the overlap between all frequency bands except the slow-5 band was found to be in the right insula (*x* = 39, *y* = 6, *z* = –21). Individual zALFF values for all frequency bands in mPFC, cerebellum/PCC, and right and left insula (*x* = –45, *y* = –18, *z* = 3) are presented in Figure 4. We also re-analyzed these data using a voxel level of *p* < 0.001 at the whole brain level. Clusters with smaller size located in the medial frontal cortex, cerebellum and the right insular cortex passed the correction threshold. The peak MNI coordinates of them were almost the same as that of clusters reported using threshold of a voxel level of *p* < 0.01 (Supplementary Materials).

### The Relationship Between Pain and Z-Score Standardized Values

**FIGURE 5:**
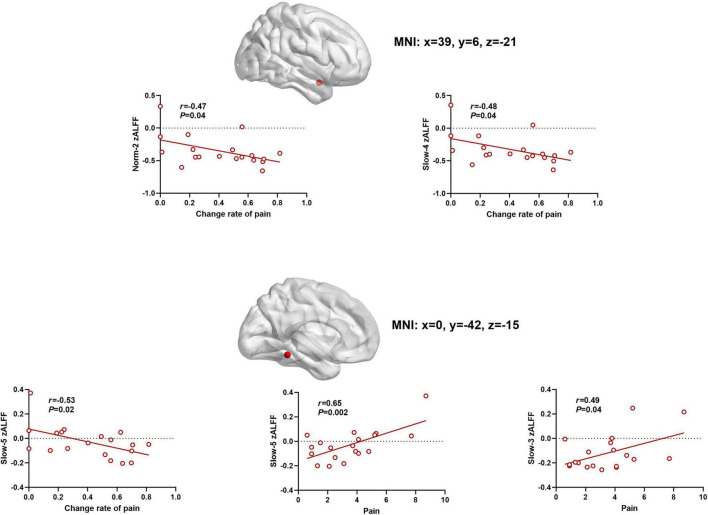
The scatter plots between zALFF and pain. Frequency-specific resting-state activity in the cerebellum and insular was correlated to AAS analgesia (the relationship were determined by Pearson coefficient value).

As shown at the top of Figure 5, there was a significant association between the above zALFF effects and VAS pain intensity scores following AAS. Specifically, slow-5 frequency band zALFF in the cerebellum was significantly positively correlated with VAS score after AAS (*r* = 0.65, *P* = 0.003), and negatively correlated with the change in VAS score between the two treatments (*r* = –0.53, *P* = 0.02). Meanwhile, slow-3 frequency band zALFF in the cerebellum was also positively correlated with VAS score after AAS (*r* = 0.48, *P* = 0.04). The zALFF within the norm-2 (*r* = –0.48, *P* = 0.04) and slow-4 (*r* = –0.48, *P* = 0.04) frequency bands in the right insula showed negative correlations with changes in VAS score after AAS.

## Discussion

AAS is a form of acupuncture therapy that does not produce any needle sensation. Its stimulation is mild and mimics a tactile sensation on the skin. Therefore, AAS may be useful in the investigation of brain responses associated with acupuncture analgesia due to the ability to exclude or minimize the confound from forms of traditional acupuncture that are associated with stimulation from Deqi needle insertion. The current study used rs-fMRI to investigate whole-brain ALFF changes following AAS, and further explored the relationship between these changes and self-reported VAS pain intensity scores. Previous functional brain imaging studies investigating acupuncture have focused its effects either in healthy subjects or in patients with a specific diagnosis. To our knowledge, the present study was the first to collect and analyze functional brain imaging data following AAS in both patients and healthy subjects. The main results showed that in contrast to tactile sham acupuncture (TSA), self-reported pain intensity in patients with CLBP significantly decreased after AAS. Meanwhile, ALFF in sub-band frequencies ranging from 0.01 to 0.25 Hz was consistently modulated within nodes of the DMN (group-level increases in the mPFC, and decreases in the cerebellum/PCC/parahippocampus) in both patients and healthy subjects. Decreased ALFF in the bilateral insular cortex (nodes of the salience network) showed frequency-specific modulations. Furthermore, pain intensity experienced by patients showed a close relationship with the functional responses within specific bands in the right insula and cerebellum.

Acupuncture stimulation induces widespread responses within DMN (Zhang et al., 2019; Zou et al., 2019). Results from studies using a within-group design indicated that the BOLD oscillations in the 0.01–0.1 Hz frequency band in the mPFC and PCC reflect acupuncture stimulation at different acupoints (including Guangming, Kunlun, and Jiaoxin) near the lateral malleolus of healthy subjects (Liu et al., 2009; Qin et al., 2011; Deng et al., 2016; Sun et al., 2019). Zhu et al. (2015) conducted a TSA-controlled (placebo-controlled) study in healthy subjects, reporting that acupuncture administered at the Taixi acupoint resulted in modulating the fractional ALFF (fALFF) in the 0.01–0.1 Hz frequency band in the form of a decrease in the mPFC and cerebellum. Further, a study by Li et al. (2014) reported a significant correlation between the modulation of functional connectivity within the DMN and the analgesic effect of acupuncture in patients with CLBP. The current study not only found similar patterns in the differential modulations of resting-state brain function between AAS and sham acupuncture in DMN but also demonstrated the consistency of the response of DMN oscillations within each frequency band in the range from 0.01 to 0.25 Hz both in healthy subjects and patients with CLBP. It is noteworthy that, compared with previous studies using manual acupuncture and electroacupuncture, which both induce a Deqi sensation, AAS, provides the absolute minimum stimulus sensation, and is still sufficient to regulate DMN function in healthy subjects and patients with chronic pain. Therefore, as argued by Otti and Noll-Hussong (2012), DMN activity involves the transformation of a variety of basic physiological or pathological body states and provides a potential biomarker for use in the study of acupuncture analgesia mechanisms, including that of AAS.

Our study provides the characteristics of multi-band BOLD signal oscillation with ALFF as the index for the central response of AAS analgesia. In present study, frequency-specific resting-state activity correlated to AAS analgesia focused in the cerebellum and insular. It is shown that plasticity changes in the insular cortex have been frequently observed in patients with chronic back pain (Baliki et al., 2012; Kim et al., 2020). Studies using structural magnetic resonance imaging have reported reduced gray matter volumes in many forebrain regions including the insula. Studies using fMRI have reported elevations in the functional synchronization of BOLD signals in the 0.12–0.2 Hz range between the insula and mPFC. A recent rs-fMRI study reported increased norm-2 band ALFF in the insular cortex associated with CLBP compared with that of healthy controls (Baliki et al., 2011; Fritz et al., 2016; Zhou et al., 2018). Results in the present and our previous research have found that decreases in ALFF across multiple frequency bands in the insula correlated with the analgesic effect of AAS (Xiang et al., 2019). This implies that the insular cortex may be an important target brain area related to the regulation of chronic pain by AAS (Xiang et al., 2019). Additionally, BOLD oscillations in the amygdala and somatosensory motor cortex have not yet been shown to correlate with the analgesic effects of AAS.

In present study, we identified frequency-specific resting-state activity in the cerebellum during the AAS stimulation. Notably, we found that norm-1 and norm-2 band ALFF patterns following acupuncture was very similar, but they did not completely overlapping, especially in the cerebellum. Only norm-2 band ALFF correlated to pain-relief effect of acupuncture. ALFF was originally defined based on norm 2 (0.01–0.08 Hz) band BOLD signal oscillation (Zang et al., 2007). However, norm 1 band oscillation was also analyzed in resting-state fMRI studies (Zuo et al., 2010). Both norm-1 and norm-2 bands are significant measures of resting-state brain activity and therefore should be considered in studies on acupuncture analgesia (Li et al., 2017; Bao et al., 2018; Wu et al., 2018). To our knowledge, this was the first study to test the potential similarity and difference between norm 1 and norm 2 in acupuncture analgesia process. The cerebellum is generally considered to be a brain region involved in motor processing. Recent researches suggest that the cerebellum has also been implicated in non-motor, and even a number of integrative functions, including memory, associative learning, motor control (Schmahmann and Pandya, 1997; Ito, 2006). Notably, some fMRI studies show activation in the cerebellum during nociceptive processing (Borsook et al., 2008). Direct evidence from electrophysiological studies indicates that the cerebellum receives nociceptive afferents (Ekerot et al., 1987; Jie and Pei-Xi, 1992). C-fiber nociceptors may act through mossy fibers to reach Purkinje cells in the cerebellum (Jie and Pei-Xi, 1992). Electrical stimulation of the intermediate portion of the anterior cerebellar lobe raised nociceptive thresholds to tail shock in monkeys (Siegel and Wepsic, 1974). Our results, consistent with other studies (Chae et al., 2013; Zheng et al., 2016; Bai et al., 2018), suggested that acupuncture stimulation modulated cerebellar activities during the process of pain relief. Current conceptualizations of pain in humans are multidimensional, mainly including the perception of the noxious stimulus, the affective features of pain, and cognitive components. Possible functional roles for the cerebellum relating to acupuncture modulation should be considered, including emotion, cognition, and motor control. Together with studies indicating a frequency-dependent modulation of BOLD signal oscillations in specific brain regions in patients with chronic pain (Wang et al., 2017; Rogachov et al., 2018; Gu et al., 2019), our study provides a practical rationale and mechanism for studying the frequency-dependence of BOLD oscillations in response to acupuncture analgesia.

In summary, the central mechanism underpinning the effects of AAS requires further explanation. This study has demonstrated a relationship between frequency-specific BOLD oscillations in the brain associated with ankle acupuncture analgesia and pain intensity scores in CLBP patients. The characteristics of BOLD signal oscillations across multiple frequency bands following AAS analgesia were demonstrated. Further work is necessary to further elucidate the mechanisms behind the effects of AAS through a combination of multi-scale, multi-modal neuroimaging, animal models, and electrophysiological techniques.

## Study Limitations

With regard to the study design and protocol, there are some limitations that need to be taken into account. Firstly, our results were observed in a small cohort of subjects. Therefore, further testing in clinical populations is warranted. Given that each different frequency band contributes uniquely to brain network integration in terms of physiological and pathological activities, the present studies of smaller size have yielded more information to help interpret localized BOLD changes associated with acupuncture analgesia and provided impetus to further investigations. These preliminary results may be of importance for the design of further confirmative studies. Secondly, washout period for AAS’s effects remains unknown, although stimulus runs were separated by structural scanning for 15 min in present study.

## Data Availability Statement

The raw data supporting the conclusions of this article will be made available by the authors, without undue reservation.

## Ethics Statement

The studies involving human participants were reviewed and approved by the Ethical Committee of the Center of Cognitive and Brain Disorders of Hangzhou Normal University. The patients/participants provided their written informed consent to participate in this study.

## Author Contributions

AX, XS, and SL conceived the study design. AX, MC, CQ, JR, and CW collected the data. AX analyzed the data. AX, MC, CQ, XS, and SL helped to draft and revise the manuscript. All authors contributed to write the article and approved the submitted version.

## Conflict of Interest

The authors declare that the research was conducted in the absence of any commercial or financial relationships that could be construed as a potential conflict of interest.

## Publisher’s Note

All claims expressed in this article are solely those of the authors and do not necessarily represent those of their affiliated organizations, or those of the publisher, the editors and the reviewers. Any product that may be evaluated in this article, or claim that may be made by its manufacturer, is not guaranteed or endorsed by the publisher.

## Footnotes

1
www.rfmri.org/dpabi

2
www.fil.ion.ucl.ac.uk/spm

3
http://restfmri.net/forum/REST_V1.3

4
www.graphpad.com/scientific-software/prism/
