# Supplementary material for: Frequency-Specific Blood Oxygen Level Dependent Oscillations Associated With Pain Relief From Ankle Acupuncture in Patients With Chronic Low Back Pain
Source: Front Neurosci. 2021 Dec 7;15:786490. doi: 10.3389/fnins.2021.786490 (PMC8688988; doi:10.3389/fnins.2021.786490)
Supplement: Supplementary file 1 [file Table_1.docx]

Supplement table1. The ALFF results of main effects (WAA-TSA)

| band | area | BA | Cluster size | *t* value | MNI coordinate | | |
| --- | --- | --- | --- | --- | --- | --- | --- |
|  |  |  |  |  | x | y | z |
| norm-1 | mPFC | 11 | 123 | 4.99 | -6 | 54 | -21 |
|  | Cerebellum | _ | 114 | -5.48 | 3 | -39 | -15 |
| norm-2 | mPFC | 11 | 124 | 5.21 | -6 | 54 | -21 |
|  | Cerebellum | _ | 106 | -5.71 | 3 | -39 | -15 |
| Slow-5 | mPFC | 11 | 75 | 5.06 | -30 | 66 | 0 |
| Slow-4 | mPFC | 11 | 159 | 5.12 | -6 | 54 | -21 |
|  | Cerebellum | _ | 58 | -5.44 | 3 | -39 | -15 |
| slow-3 | mPFC | 11 | 205 | 6.10 | 0 | 39 | -24 |
|  | Cerebellum | _ | 67 | -4.64 | -15 | -36 | -18 |
| slow-2 | mPFC | 11 | 137 | 5.16 | 12 | 36 | -24 |
|  | Cerebellum | _ | 117 | -5.33 | 6 | -48 | -42 |
|  |  | _ | 153 | -5.43 | 15 | -36 | -15 |
|  | Right insula, amygdala | 13, 22 | 180 | -5.12 | 45 | 0 | 3 |

（BA=Brodmann，MNI= Montreal Neurological Institute, mPFC=medial prefrontal cortex,）


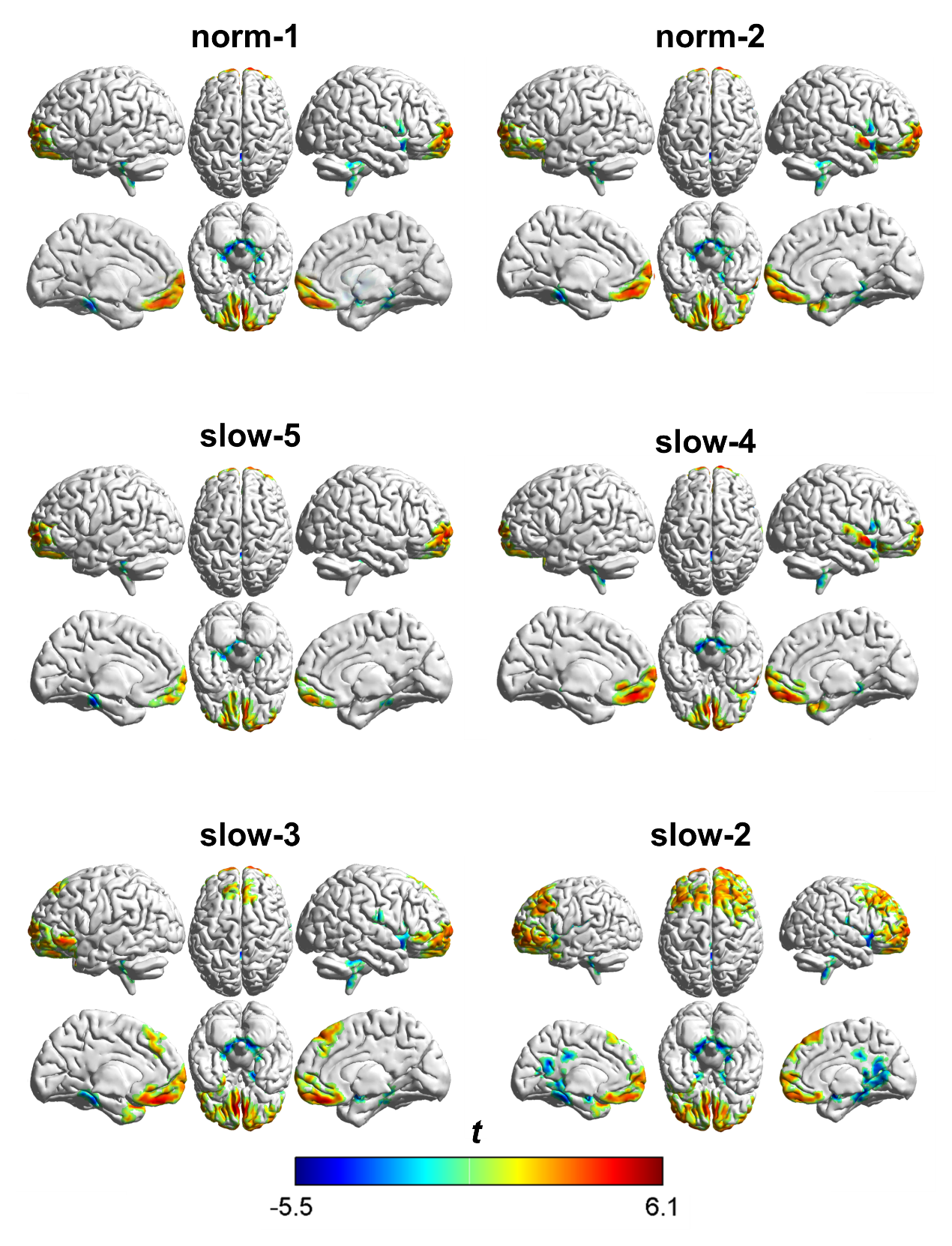


Supplementary figure1. The ALFF map of main effects (WAA-TSA)
